# Identification of biomarkers and potential drug targets in DFU based on fundamental experiments and multi-omics joint analysis

**DOI:** 10.3389/fphar.2025.1561179

**Published:** 2025-05-23

**Authors:** Xudong Xin, Haidong Zhou, Song Huang, Wenzhao Zhang, Jiahou Xu, Wei Wang, Jihua Wei, Liqing Li

**Affiliations:** ^1^ Affiliated Hospital of Youjiang Medical University for Nationalities, Baise, Guangxi, China; ^2^ Guangxi Zhuang Autonomous Region Engineering Research Center for Biomaterials in Bone and Joint Degenerative Diseases, Baise, Guangxi, China; ^3^ Guangxi Key Laboratory for Preclinical and Translational Research on Bone and Joint Degenerative Diseases, Baise, Guangxi, China; ^4^ Guangxi Key Laboratory of Clinical Cohort Research on Bone and Joint Degenerative Disease, Baise, Guangxi, China; ^5^ Youjiang Medical University for Nationalities, Baise, Guangxi, China; ^6^ The Central Research Laboratory, Hunan Traditional Chinese Medical College, Zhuzhou, Hunan, China

**Keywords:** macrophage, diabetic foot ulcers, single cells, bioinformatics, quercetin, molecular docking

## Abstract

**Objective:**

This study aims to investigate the molecular mechanisms by which quercetin facilitates the treatment of diabetic foot ulcers (DFU).

**Methods:**

Transcriptome sequencing datasets for DFU, specifically GSE80178, GSE134431, and GSE147890, along with single-cell dataset GSE165816, were retrieved from the Gene Expression Omnibus (GEO) online database (https://www.ncbi.nlm.nih.gov/geo/). The single-cell data were subjected to processing, annotation, differential gene expression analysis, and staining. The transcriptome sequencing data were analyzed using weighted gene co-expression network analysis (WGCNA), followed by assessment of immune infiltration. By integrating transcriptomic data and differentially expressed genes identified through WGCNA, co-expressed differentially expressed genes were obtained, and a protein-protein interaction (PPI) network was constructed followed by enrichment analysis. Core genes were screened using four machine learning models (Random Forest, Lasso, XGBoost, and SVM). Drug prediction was performed to identify potential therapeutic agents, and molecular docking simulations were conducted to assess the binding interactions between the macromolecular proteins encoded by the core genes and quercetin. A rat model of diabetic foot ulcer (DFU) was established and randomly divided into three groups: control, model, and treatment groups. Tissue samples were collected at 3, 7, and 14 days post-intervention for RT-qPCR, hematoxylin and eosin (H&E) staining, Masson’s trichrome staining, and immunofluorescence staining to evaluate the therapeutic effects of quercetin via modulation of the core genes on DFU.

**Results:**

The analysis identified 275 differentially co-expressed genes that are extensively involved in the IL-17 signaling pathway, metabolic pathways, the PI3K/Akt signaling pathway, *Staphylococcus aureus* infection, complement and coagulation cascades, among others. From these, four core genes (CIB2, SAMHD1, DPYSL2, IFI44) were selected using machine learning techniques. Immune infiltration analysis demonstrated a strong correlation between SAMHD1, IFI44, DPYSL2, and macrophages. Molecular docking studies revealed that quercetin exhibits a lower binding energy with the target protein binding site, forming a stable structure. Single-cell analysis indicated that SAMHD1 is predominantly expressed in macrophages, whereas DPYSL2 is expressed not only in macrophages but also significantly in vascular endothelial cells and other cell types. This suggests that SAMHD1 and DPYSL2 may exert their effects by modulating these cells, as corroborated by basic experimental findings. The improvement in wound tissue morphology observed in the treatment group was more favorable compared to the model group. In comparison to the acute group, the gene expression profile in the model group aligned with bioinformatics predictions. Furthermore, the alterations in core gene expression following quercetin treatment were statistically significant.

**Conclusion:**

Quercetin may enhance the healing of diabetic foot ulcers by modulating macrophage activity through the regulation of SAMHD1 and DPYSL2, thereby contributing to the recovery process.

## 1 Introduction

Diabetes is widely acknowledged as one of the most critical global health challenges. Currently, over 500 million adults worldwide are affected by diabetes, with approximately 10% of the global population being diabetic. Projections indicate that by 2030, this figure will increase to 643 million. Among the most severe complications of diabetes is diabetic foot, which can result in circulatory and sensory impairments in the patient’s feet. Notably, around 20% of patients with diabetic foot ulcers (DFUs) require lower limb amputation (Armstrong et al., 2017), significantly diminishing the quality of life for individuals with advanced diabetes. Quercetin, a flavonoid, exhibits promising anti-diabetic (Dhanya, 2022), anti-inflammatory (Yuan et al., 2020), and blood circulation-enhancing properties (Larson et al., 2010). Additionally, flavonoids have been demonstrated to aid in the prevention of neurodegenerative diseases and may delay neurodegeneration processes (Khan et al., 2019). This suggests that quercetin treatment could potentially address diabetic foot ulcers through multiple mechanisms. However, the precise mechanism by which quercetin modulates macrophages to facilitate wound healing in diabetic foot conditions remains unclear. This study seeks to investigate the effects of quercetin on wound healing in diabetic wounds through multi-omics integration and analysis, alongside fundamental experiments, to elucidate its mechanisms.

## 2 Methods

### 2.1 Bioinformatics

#### 2.1.1 Download raw data

GEO (http://www.ncbi.nlm.nih.gov/geo) (Edgar et al., 2002) is a public database that contains a large number of sequencing results submitted by research institutions from around the world. Transcriptome data on diabetic foot ulcers (GSE134431 (Sawaya et al., 2020), GSE80178 (Ramirez et al., 2018), GSE147890 (Leon et al., 2020)) and single-cell data (GSE223964 (Chen et al., 2024)).

#### 2.1.2 Weighted gene co-expression network analysis

In the Weighted Gene Co-expression Network Analysis (WGCNA), the topology calculation employs a soft threshold power ranging from 1 to 20. Utilizing the optimal soft threshold power (*β* = 19), the correlation matrix is transformed into an adjacency matrix, which is subsequently converted into a topological overlap matrix (TOM). The TOM is then used to conduct average linkage hierarchical clustering on modules comprising a minimum of 300 genes. Following this, similar modules are merged based on a cutting height of 0.25. The Pearson method is employed to compute the correlation between the combined modules and the incidence of diabetic foot ulcers, leading to the identification of the module with the highest correlation as the core module. The genes within this core module will be utilized in the subsequent phase of the analysis.

#### 2.1.3 Differential expression visualization

Integrate the transcriptome datasets (GSE134431, GSE80178, and GSE147890) and conduct batch effect correction, normalization, and standardization. Differential gene expression (DEG) selection criteria are set at |log2FC| > 1 and an adjusted *p*-value <0.05. Utilize the DAVID online platform (https://david.ncifcrf.gov/) to perform Gene Ontology (GO) and KEGG pathway analyses on the common differential genes identified through Weighted Gene Co-expression Network Analysis (WGCNA) and transcriptome data (Kanehisa et al., 2023; Kanehisa, 2019). Employ the Microbioinformatics online platform (http://www.bioinformatics.com.cn/) to visualize the common DEGs, producing heatmaps, volcano plots, Venn diagrams, and bubble plots.

#### 2.1.4 Machine learning

The dataset was partitioned into a training set comprising 70% of the data and a test set comprising 30%. The Random Forest machine learning model was implemented using the randomForest package in R to analyze the predictor variables. A 10-fold cross-validation approach was employed to train the model, resulting in the identification of the top 15 genes as hub genes based on their importance ranking. Predictions on the test set were conducted using LASSO regression, SGBoost, and SVM algorithms to further pinpoint key genes influencing patient prognosis, also utilizing 10-fold cross-validation.

#### 2.1.5 Core gene validation

The model’s diagnostic performance was evaluated using the Receiver Operating Characteristic (ROC) curve and the Area Under the Curve (AUC) metric. To verify the transcription levels of core genes, a *t*-test was conducted on the original datasets (GSE134431, GSE80178, and GSE147890) using SPSS 26.0 software, with a *P*-value of less than 0.05 considered statistically significant. Additionally, ROC analysis of the hub genes was performed using the Xiantao Academic Online Analysis Platform (https://www.xiantaozi.com/).

#### 2.1.6 Immune infiltration analysis

The CIBERSORT algorithm is employed to analyze gene expression matrices and estimate the subpopulations of infiltrating immune cells within samples. Samples are filtered using an adjusted P-value threshold of less than 0.05, and the outputs of the MOABS algorithm, along with the immune cell infiltration matrix, are calculated. The R package “GSVA” is utilized to conduct single-sample Gene Set Enrichment Analysis (ssGSEA) on the disease group. Furthermore, the R package “pheatmap” (available at https://CRAN.R-project.org/package=pheatmap) is used to visualize the correlations between samples.

#### 2.1.7 Single-cell genomics

Single-cell data were obtained from the Gene Expression Omnibus database (accession number GSE223964), comprising eight samples from patients with diabetic foot ulcers (DFU). The data were processed and analyzed using the Seurat R package, resulting in the creation of Seurat objects. Quality control parameters were established, including the expression of genes per cell ranging from 200 to 5,000, and mitochondrial gene expression was restricted to less than 8%. Subsequently, the Seurat object was normalized, and highly variable genes along with cell cycle scores were computed. The RunHarmony command was employed to mitigate batch effects. For dimensionality reduction, principal component analysis (PCA) and uniform manifold approximation and projection (UMAP) were utilized to reduce the dimensionality of the highly variable genes. Unsupervised clustering of the cells was performed using the FindClusters command with a resolution parameter set to 0.2. Based on established cell-specific markers, these clusters were annotated into 13 distinct cell types. The distribution of hub genes across different cell types was visualized using cell staining techniques and horn plots.

#### 2.1.8 Drug prediction and molecular docking experiments

Utilize Enrichr (available at https://maayanlab.cloud/Enrichr/) (Kuleshov et al., 2016) for the analysis of DSigDB. The crystal structures of the relevant key proteins can be retrieved from the Protein Data Bank (accessible at https://www.rcsb.org/). All molecular docking experiments were performed using the Autodock software, version 1.5.7. The outcomes are reported in terms of binding energy. The final visualization is generated using the Pymol plugin for Python 37.

### 2.2 Basic experiment

#### 2.2.1 Preliminary preparations

##### 2.2.1.1 Animal origin

SPF-grade Wistar male rats, 30 individuals, 12 weeks old, body weight 220–250 g, produced by Changsha Tianqin Biotechnology Co., Ltd.

#### 2.2.2 Experimental methods

##### 2.2.2.1 Experimental grouping and modeling

Following a 1-week acclimatization period in a specific pathogen-free (SPF) laboratory environment, 30 SPF-grade Sprague-Dawley male rats were randomly allocated into three groups: an acute group (*n* = 10), a model group (*n* = 10), and a treatment group (*n* = 10). To establish a rat model of type 1 diabetes, the rats were fasted for 12 h with unrestricted access to water. On the day of model induction, the rats were weighed, and blood samples were collected from the tail vein to determine baseline blood glucose levels using a glucometer. Streptozotocin was dissolved in a 0.1 mol/L citric acid-sodium citrate buffer solution at pH 4.2 to create a 1% solution. The rats of model group and treatment group were induced with diabetes mellitus via a single intraperitoneal injection of STZ at a dose of 100 mg/kg body weight, whereas the control group rats received an equivalent volume of the citric acid-sodium citrate buffer solution via intraperitoneal injection. Seventy-two hours following the administration of streptozotocin (STZ), fasting blood glucose levels should be assessed using blood obtained from the tail vein. Rats exhibiting blood glucose levels exceeding 16.7 mmol/L, along with characteristic symptoms such as polydipsia, hyperphagia, polyuria, and weight loss, are classified as diabetic mellitus (DM) rats. Weekly assessments of the rats’ body weight and blood glucose levels should be conducted. To establish the diabetic foot ulcer (DFU) model, once blood glucose levels in the diabetic rats have stabilized, the rats should be anesthetized with ketamine (75 mg/kg, intraperitoneally) and thioridazine (10 mg/kg, intraperitoneally). Subsequently, near the popliteal fossa, the femoral artery should be ligated, and a soft transparent plastic template should be used to create a rectangular wound measuring 1 cm by 2 cm on the dorsum of each rat’s foot, thereby establishing an acute ischemic DFU model.

##### 2.2.2.2 Intervention therapy

Following successful modeling, the rats in the treatment group underwent wound cleaning with a 1/5000 furacin solution and had their dressings changed every 8–12 h using quercetin dissolved in DMSO, which was applied directly to the wound. In contrast, the control groups received wound cleaning with the same furacin solution and a saline application.

##### 2.2.2.3 Specimen collection

On days 3, 7, and 14 post-modeling, 3, 3, and 4 rats were randomly selected from each group, respectively. After anesthesia, the entire wound along with the surrounding tissue, extending 0.5 cm from the wound edge, was excised down to the fascia layer. A portion of the excised tissue was stored at 4°C for histological examination via pathological sections. The remaining tissue samples were temporarily placed in liquid nitrogen and then promptly transferred to a −80°C freezer. Upon completion of sample collection at the three designated time points, all samples were uniformly subjected to reverse transcription quantitative polymerase chain reaction (RT-qPCR) detection and analysis.

##### 2.2.2.4 Observe wound healing

Systematically monitor the wound healing process in rats across different groups by documenting growth conditions and capturing images of the wounds at standardized focal lengths on days 3, 7, and 14 post-intervention. This approach facilitates a comparative analysis of the wound healing progression and condition at each time point within each group.

##### 2.2.2.5 Observation of organizational forms

Employ hematoxylin-eosin (HE) staining to assess pathological morphological changes: dehydrate the wound tissue, clear it, embed it in paraffin, section the paraffin block, mount, adhere, and bake the sections, followed by deparaffinization, hydration, and HE staining. Examine the sections under a biological microscope to observe morphological changes within each tissue group and capture images. Utilize Masson’s Trichrome Stain to evaluate alterations in collagen growth during skin wound healing: replicate the dehydration to hydration steps as in HE staining, then proceed with Masson staining. Observe under a biological microscope, capture images, and analyze collagen volume using ImageJ software.

##### 2.2.2.6 Real-time quantitative PCR (RT qPCR) method

Total RNA was extracted from tissue samples, followed by RNA electrophoresis and reverse transcription using the TIANScript RT Kit. The mRNA expression levels of CIB2, IFI44, DPYSL2, and SAMHD1 were subsequently quantified using the RT-qPCR method. A fluorescent quantitative PCR instrument was employed to determine the cycle threshold (Ct) values through specialized software. Relative quantification of the gene expression data was conducted using the 2^ ^−△△^CT method.

##### 2.2.2.7 Immunofluorescence staining

The tissue was fixed in 10% formaldehyde in phosphate-buffered saline (PBS) for 10 min, followed by three rinses with PBS. Subsequently, the tissue was incubated at room temperature for 1 h in a solution containing 5% normal serum and 0.25% Triton X-100 in PBS. The tissue was then incubated overnight at 4°C with primary antibodies against CIB2, DPYSL2, IFI44, and SAMHD1 at a dilution of 1:200. After three additional rinses with PBS, each lasting 5 min, the tissue was exposed to an Alexa Fluor 488-conjugated goat anti-rabbit antibody at a dilution of 1:1000 for 20 min. For nuclear staining, 4′,6-diamidino-2-phenylindole (DAPI) was applied, and imaging was performed using a confocal scanning microscope. Fluorescence intensity of CIB2, DPYSL2, IFI44, and SAMHD1 was subsequently analyzed using ImageJ software.

##### 2.2.2.8 Statistical methods

Statistical analyses were conducted using SPSS version 26.0 to represent measurement data and to compare multiple samples through one-way analysis of variance (ANOVA). Based on the results of the homogeneity of variance test, the Least Significant Difference (LSD) method was employed when variances were equal, while the Tamhane’s T2 method was utilized in cases of unequal variances. A *p*-value of less than 0.05 was considered indicative of statistical significance.

## 3 Result

### 3.1 Weighted gene co-expression network analysis

**FIGURE 1:**
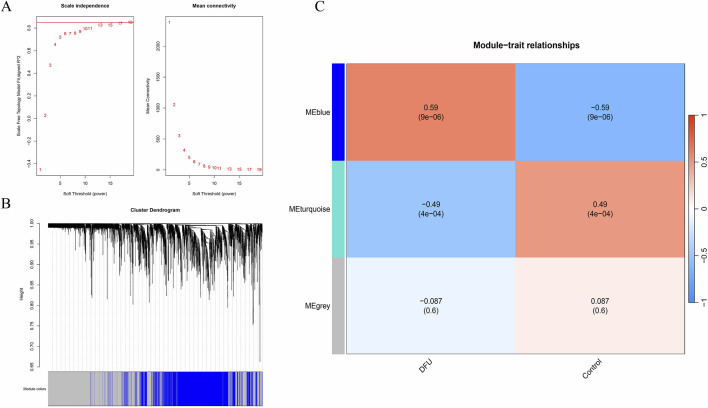
WGCNA. **(A)** Soft threshold; **(B)** Gene clustering tree; **(C)** Clinical trait correlation module.

To elucidate the relationship between clinical data and key genes, we performed a Weighted Gene Co-expression Network Analysis (WGCNA). We executed topological calculations within a soft threshold power range of 1–20, identifying an optimal soft threshold power of 19 (Figure 1A). Utilizing this soft threshold, we transformed the correlation matrix into an adjacency matrix, which was subsequently converted into a Topological Overlap Matrix (TOM). Through average linkage hierarchical clustering based on the TOM, we classified relevant gene modules, ensuring each module comprised a minimum of 300 genes (Figure 1B). We then merged similar gene modules to identify three distinct modules (Figure 1C). Additionally, we calculated the correlation between module-specific genes and clinical characteristics. Notably, the blue module, encompassing 1,477 genes, demonstrated the highest significance concerning the incidence of diabetic foot ulcers (DFU), with a *P*-value of 9e-06. Therefore, the genes in the blue module were selected for subsequent analysis.

### 3.2 Differential expression visualization

**FIGURE 2:**
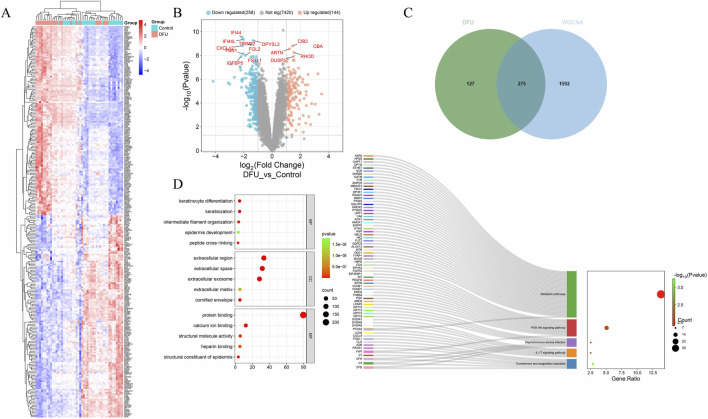
Differential expression visualization. **(A)** Differential gene heatmap; **(B)** Volcano plot; **(C)** Venn diagram; **(D)** GO and KEGG enrichment analysis.

By intersecting differentially expressed genes identified through Weighted Gene Co-expression Network Analysis (WGCNA) and transcriptomic analysis, we identified 275 common differentially expressed genes (DEGs) (Figure 2C). Subsequently, we generated heatmaps (Figure 2A) and volcano plots (Figure 2B) to visualize these common DEGs. To explore the biological functions and pathways associated with these DEGs, we performed Gene Ontology (GO) and Kyoto Encyclopedia of Genes and Genomes (KEGG) pathway enrichment analyses (Figure 2D). After applying an adjusted p-value threshold of <0.05, we selected the top five significantly enriched GO terms and the top five KEGG pathways. In terms of biological processes, the common DEGs are predominantly associated with keratinocyte differentiation, keratinization, peptide cross-linking, intermediate filament organization, and epidermal development. The analysis of cellular components reveals a significant enrichment of common differential genes within the extracellular space, extracellular region, exosomes, keratinized envelope, and extracellular matrix. The molecular function analysis indicates that these genes are predominantly enriched in epidermal structural components, protein binding, calcium ion binding, structural molecule activity, and heparin binding. Furthermore, the KEGG pathway analysis demonstrates that the common differential genes are significantly associated with pathways such as complement and coagulation cascades, IL-17 signaling pathway, *Staphylococcus aureus* infection, PI3K-Akt signaling pathway, metabolic pathways, among others.

### 3.3 Machine learning and core gene validation

**FIGURE 3:**
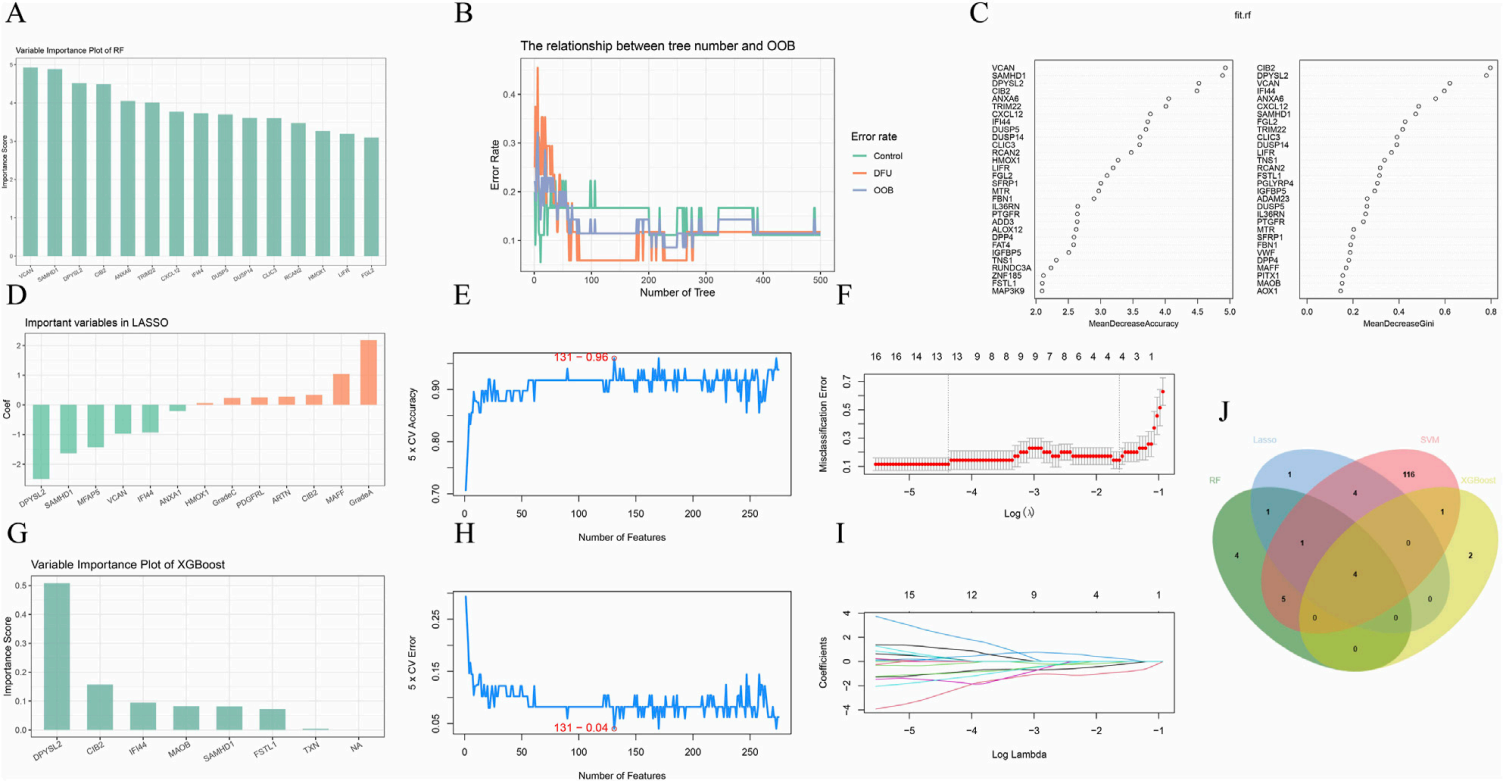
Machine learning. **(A)** Hub genes selected by Random Forest; **(B)** RF algorithm; **(C)** RF variable importance; **(D)** Hub genes selected by Lasso; **(E,H)** SVM-REF algorithm; **(F,I)** Lasso algorithm; **(G)** Hub genes selected by XGBoost; **(J)** Venn diagram of four machine learning algorithms.

**FIGURE 4:**
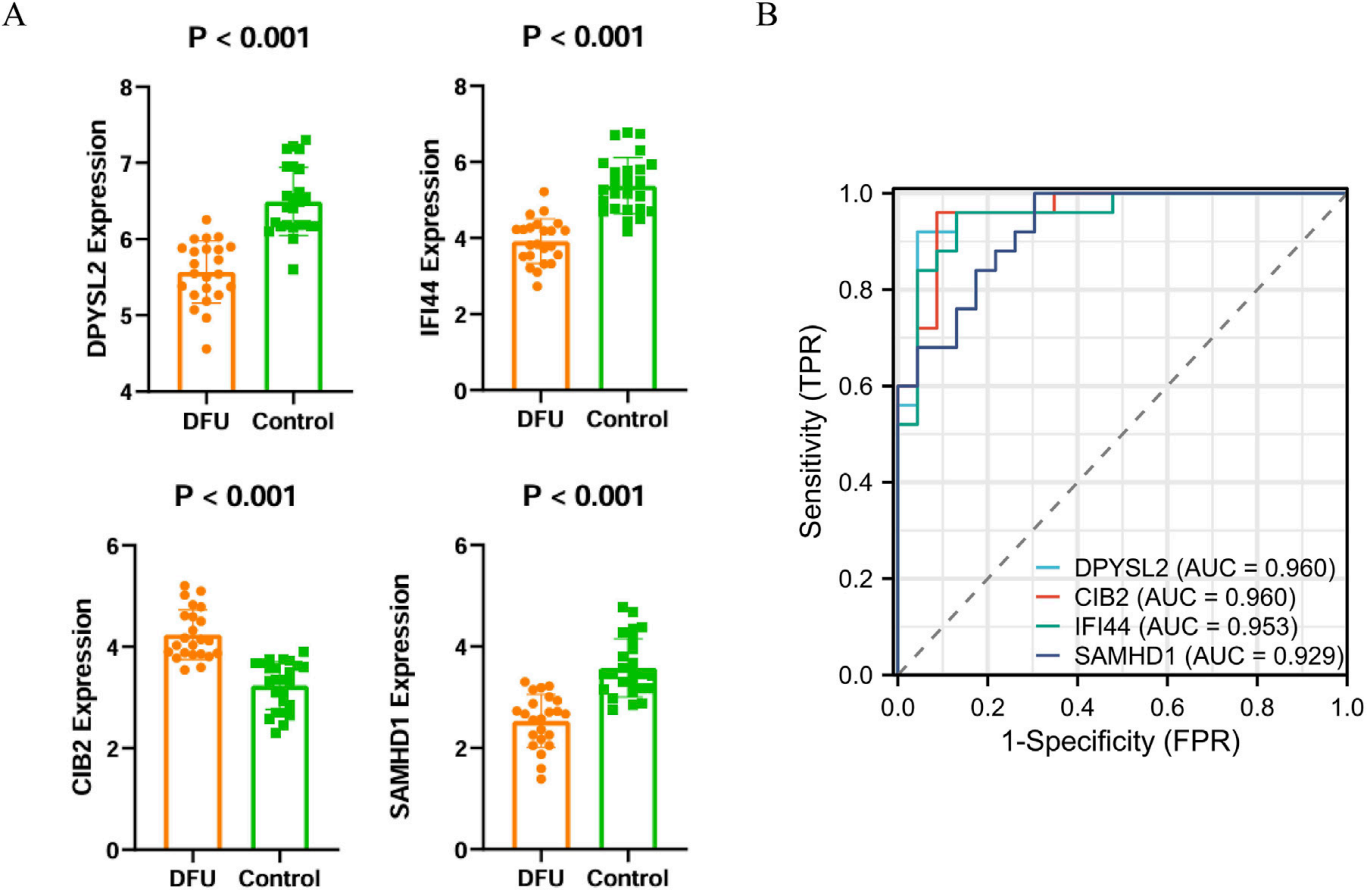
Validation of core genes and ROC diagnostic analysis. **(A)** Bar chart of core gene expression levels; **(B)** ROC curve of core genes.

To identify disease-related core genes, we developed four algorithmic models: Random Forest (Figures 3A–C), Lasso (Figures 3D,F,I), XGBoost (Figure 3G), and Support Vector Machine (SVM) (Figures 3E,H). By integrating the outcomes of these models (Figure 3J), we identified the hub genes: SAMHD1, DPYSL2, CIB2, and IFI44. To assess the accuracy of the differentially expressed genes, we conducted a *t*-test using SPSS version 26.0. The results demonstrated that the *P*-values for SAMHD1, DPYSL2, CIB2, and IFI44 were all less than 0.001 (Figure 4A), indicating statistically significant differences. Subsequently, we performed Receiver Operating Characteristic (ROC) analysis on the differentially expressed genes (Figure 4B). The analysis revealed that the Area Under the Curve (AUC) for SAMHD1 was 0.929, for DPYSL2 was 0.96, for CIB2 was 0.96, and for IFI44 was 0.953, thereby confirming that SAMHD1, DPYSL2, CIB2, and IFI44 exhibit considerable diagnostic potential.

### 3.4 Immune infiltration analysis

**FIGURE 5:**
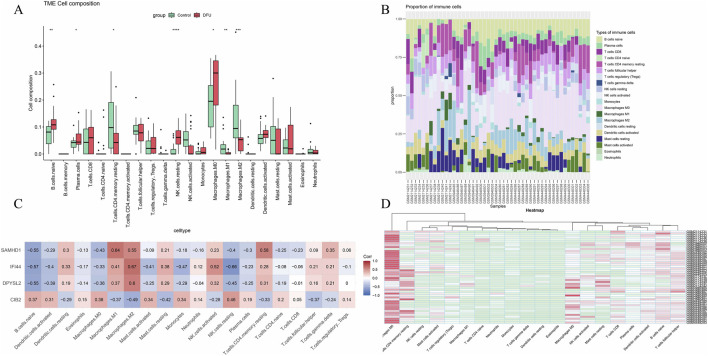
Immune infiltration analysis. **(A)** Box plot of immune cell infiltration expression in the disease group versus the control group; **(B)** Relative abundance of different immune cell subsets in diabetic foot ulcers; **(C)** Heatmap of the correlation between core genes and immune infiltration; **(D)** Heatmap of immune infiltration correlation across various datasets.

Inflammation is a pivotal factor in the onset and progression of diabetic foot ulcers (DFU). To assess the level of inflammation, the CIBERSORT algorithm was utilized to estimate immune cell infiltration in patients with DFU. The analysis of the relative abundance of various immune cell subsets in DFU identified B cells, plasma cells, CD4 T cells, natural killer (NK) cells, and macrophages as the predominant infiltrating immune cell types (Figure 5A). Within the cohort of M1 macrophages, a positive correlation with SAMHD1 was observed; similarly, in the M2 macrophage cohort, SAMHD1, IFI44, and DPYSL2 were positively correlated (Figure 5C). Correlation coefficient analysis in DFU revealed a significant association between core genes and immune cell infiltration levels, suggesting a link between DFU and immune infiltration.

### 3.5 Single-cell annotation and enrichment analysis

**FIGURE 6:**
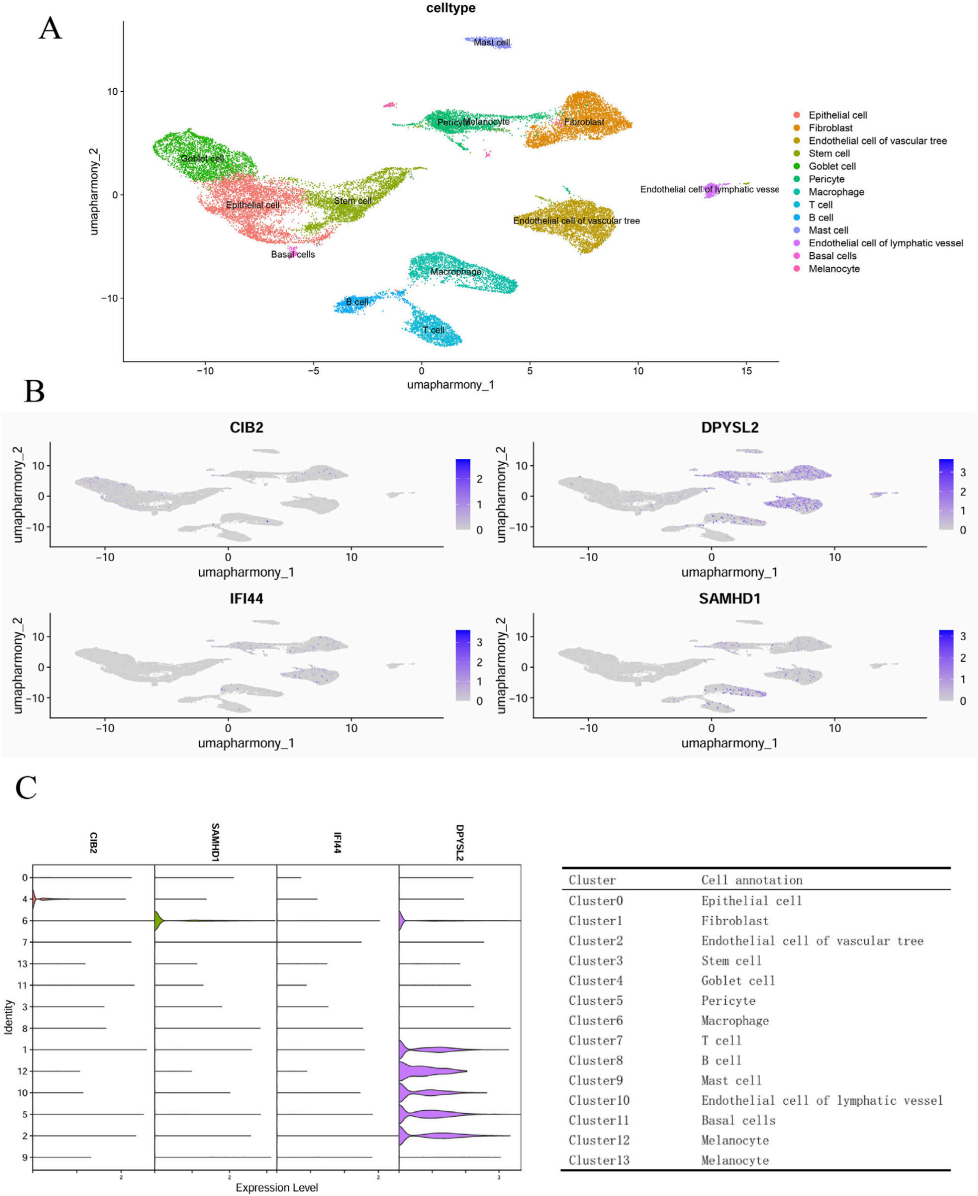
Single-cell genomics. **(A)** Cell annotation; **(B)** Cell staining; **(C)** Hub gene funnel plot and cluster annotation.

In the GSE165816 dataset, we selected foot tissue samples from eight patients diagnosed with diabetic foot ulcers (DFU). Following this, we undertook data normalization, filtered for highly variable genes, performed dimensionality reduction, and addressed batch effects. Further dimensionality reduction was achieved using UMAP, and graph-based clustering was employed, resulting in the identification of 13 distinct cell clusters. Annotation of these clusters was performed utilizing the ACT online database (http://xteam.xbio.top/ACT/) and the PanglaoDB database (https://panglaodb.se/#google_vignette). This analysis revealed the presence of epithelial cells, fibroblasts, vascular endothelial cells, stem cells, goblet cells, pericytes, macrophages, T cells, B cells, mast cells, lymphatic endothelial cells, basal cells, and melanocytes (Figure 6A). To explore the expression of core genes within specific cell types, we conducted cellular staining (Figure 6B) and generated expression funnel plots (Figure 6C) for the samples. The findings indicated that DPYSL2 and SAMHD1 are predominantly expressed in macrophages, suggesting a potential role in macrophage regulation.

### 3.6 Drug prediction and molecular docking experiments

**FIGURE 7:**
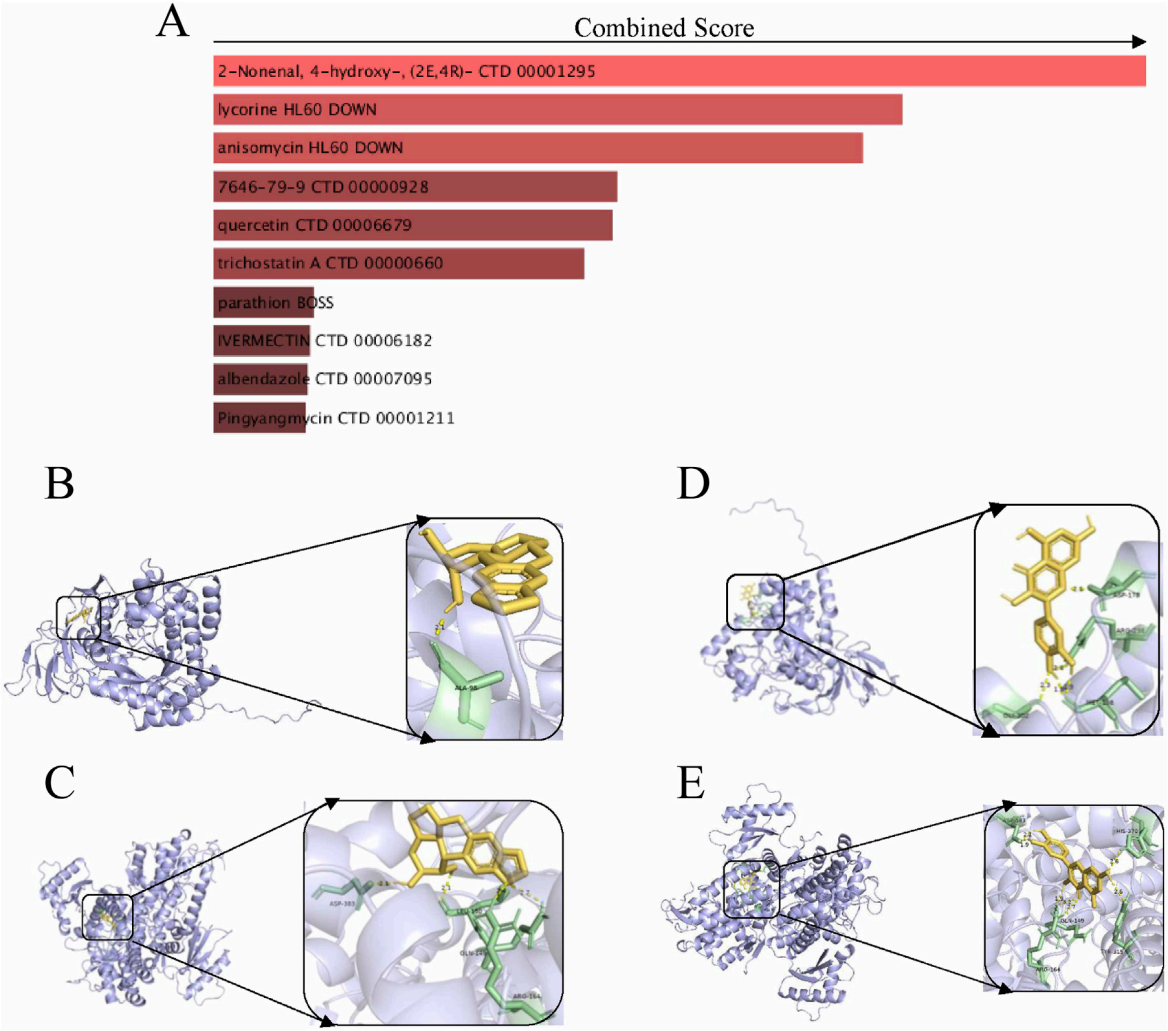
Drug prediction and molecular docking experiments. **(A)** Drug prediction; **(B)** Molecular docking of lycorine with DPYSL2; **(C)** Molecular docking of lycorine with SAMHD1; **(D)** Molecular docking of quercetin with DPYSL2; **(E)** Molecular docking of quercetin with SAMHD1.

The chemical-protein interaction network serves as an essential research tool for elucidating protein functions and facilitating advancements in drug discovery. Utilizing enrichment analysis from the DSigDB database, we focused on the core genes associated with diabetic foot ulcers (DFU), specifically SAMHD1 and DPYSL2, to identify potential drug candidates. The top 10 drug molecules were selected based on their binding affinities, with the top five candidates being 2-nonenal, 4-hydroxy-, lycorine, anisomycin, cobalt chloride, and quercetin (Figure 7A). Molecular docking analyses were conducted to predict the interactions of lycorine and quercetin with SAMHD1 and DPYSL2. The findings (Figures 7B–D) indicated that quercetin exhibited lower stabilization energy at the binding sites of the target proteins and formed a stable complex. The binding energy, along with the number and positions of hydrogen bonds formed between quercetin and SAMHD1/DPYSL2, were assessed using AutoDock calculations. Consequently, quercetin may possess potential therapeutic effects for the treatment of DFU.

### 3.7 Observe wound healing

**FIGURE 8:**
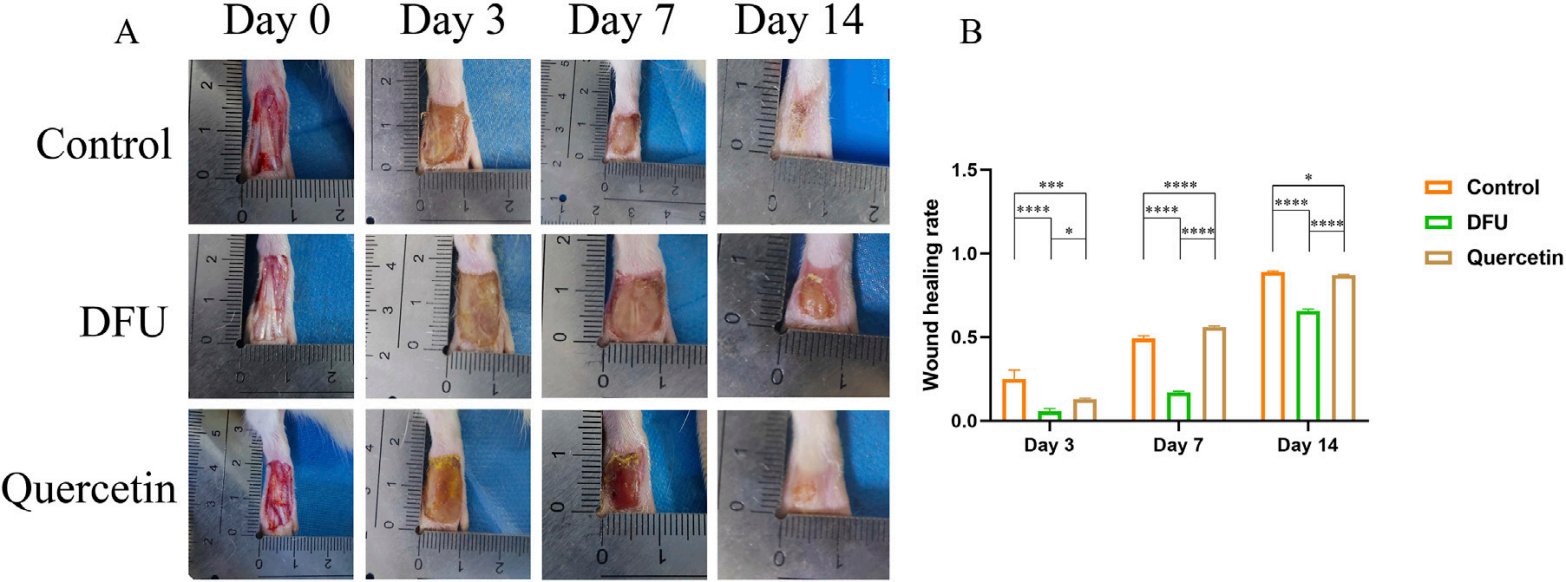
Wound healing (*P < 0.05, **P < 0.01, ***P < 0.005, and ****P < 0.001). **(A)** Wound healing; **(B)** Wound healing rate.

Following the modeling procedure, all rats maintained normal dietary habits, water consumption, and activity levels. By the third and seventh days post-modeling, the model group displayed yellow purulent deposits on their wounds. In contrast, the acute and treatment groups exhibited no significant tissue edema or purulent discharge from the early to mid-stages of wound healing. By the 14th day, the wound area in the model group remained larger than that observed in the other three groups. The wounds in the control group were almost completely healed, while those in the treatment group were mostly healed, with substantial new hair growth surrounding the wound perimeters (Figure 8A). Statistical analysis conducted using ImageJ indicated that wound healing was delayed in the model group, whereas the control and treatment groups showed superior healing outcomes compared to the model group (Figure 8B).

### 3.8 RT-qPCR

**FIGURE 9:**
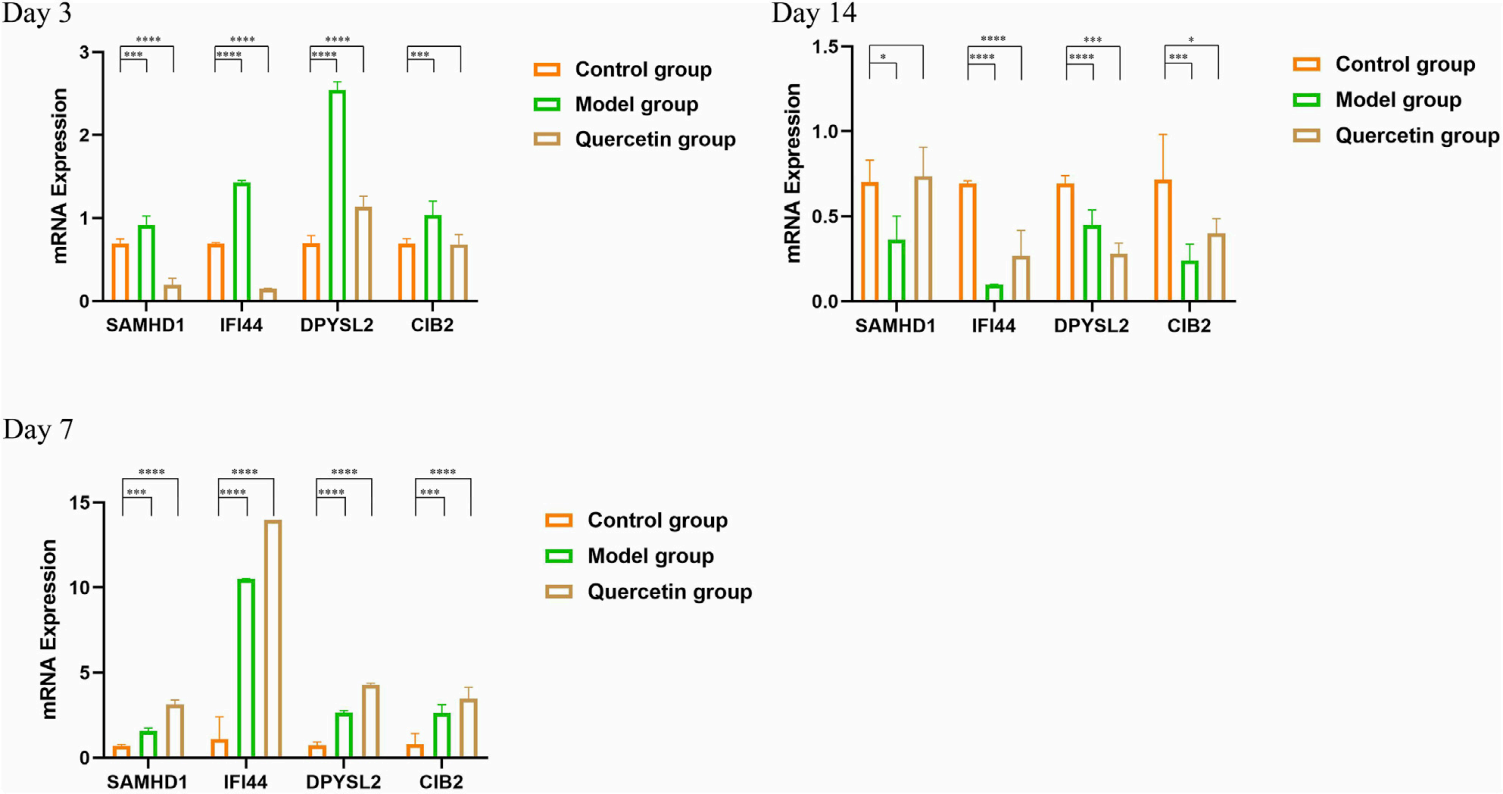
RT-qPCR (*P < 0.05, **P < 0.01, ***P < 0.005, and ****P < 0.001).

On the third day following modeling and intervention treatment, the model group exhibited a statistically significant increase in the expression levels of all core genes compared to the control group at the corresponding time point (*P* < 0.001). Conversely, in the treatment group, the expression levels of SAMHD1 and IFI44 were significantly decreased and the expression of DPYSL2 increased (*P* < 0.001). By the seventh day post-intervention, the model group continued to show a significant elevation in the expression levels of all core genes relative to the control group (*P* < 0.005). In the treatment group, the expression levels of all core genes were similarly increased, with statistical significance (*P* < 0.001). On the 14th day after modeling and intervention, the model group demonstrated a statistically significant reduction in the expression levels of all core genes compared to the control group (*P* < 0.05). In the treatment group, the expression levels of IFI44, DPYSL2, and CIB2 were also significantly decreased (*P* < 0.05) (Figure 9).

### 3.9 The wound tissue was stained morphologically

**FIGURE 10:**
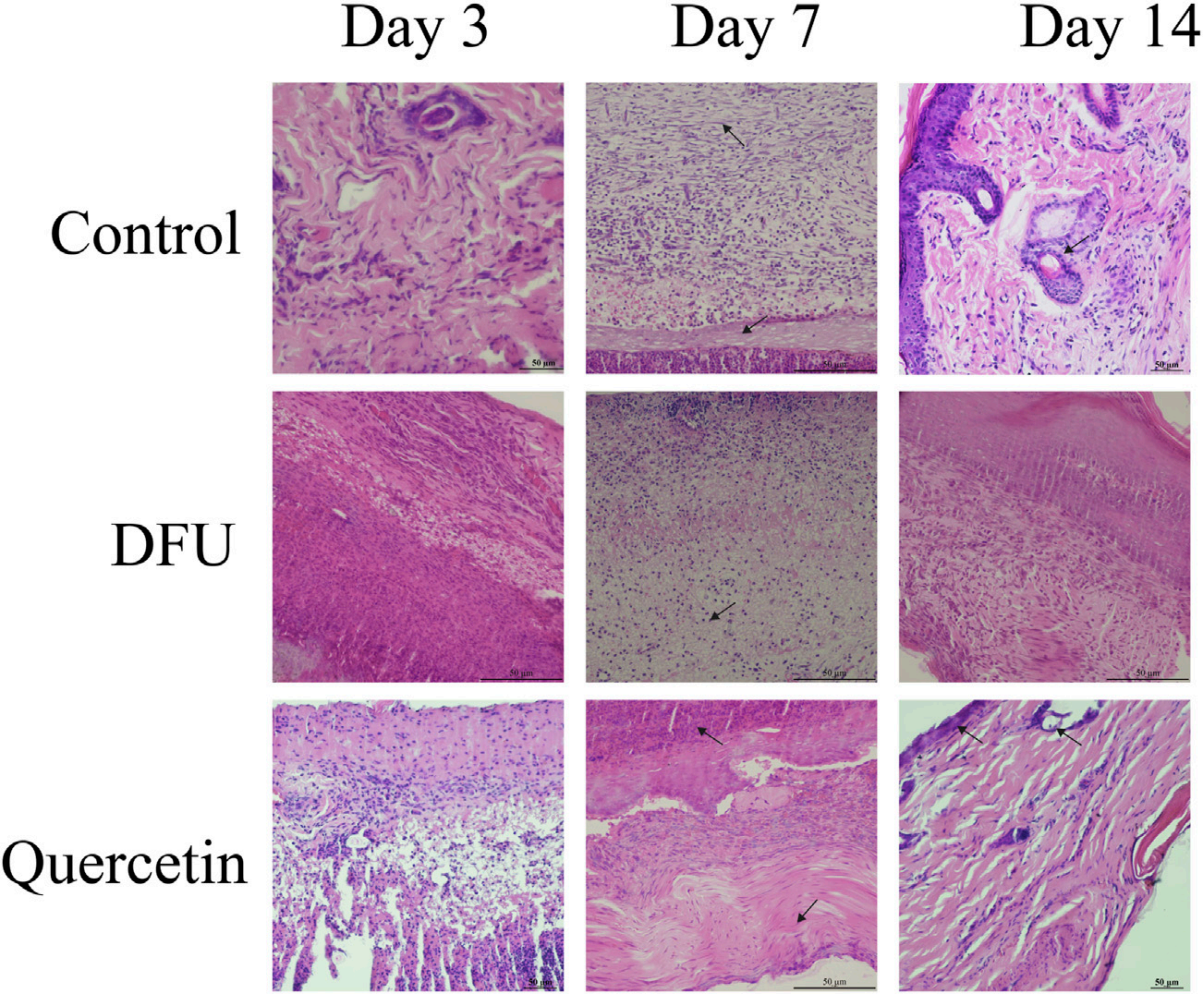
HE staining.

**FIGURE 11:**
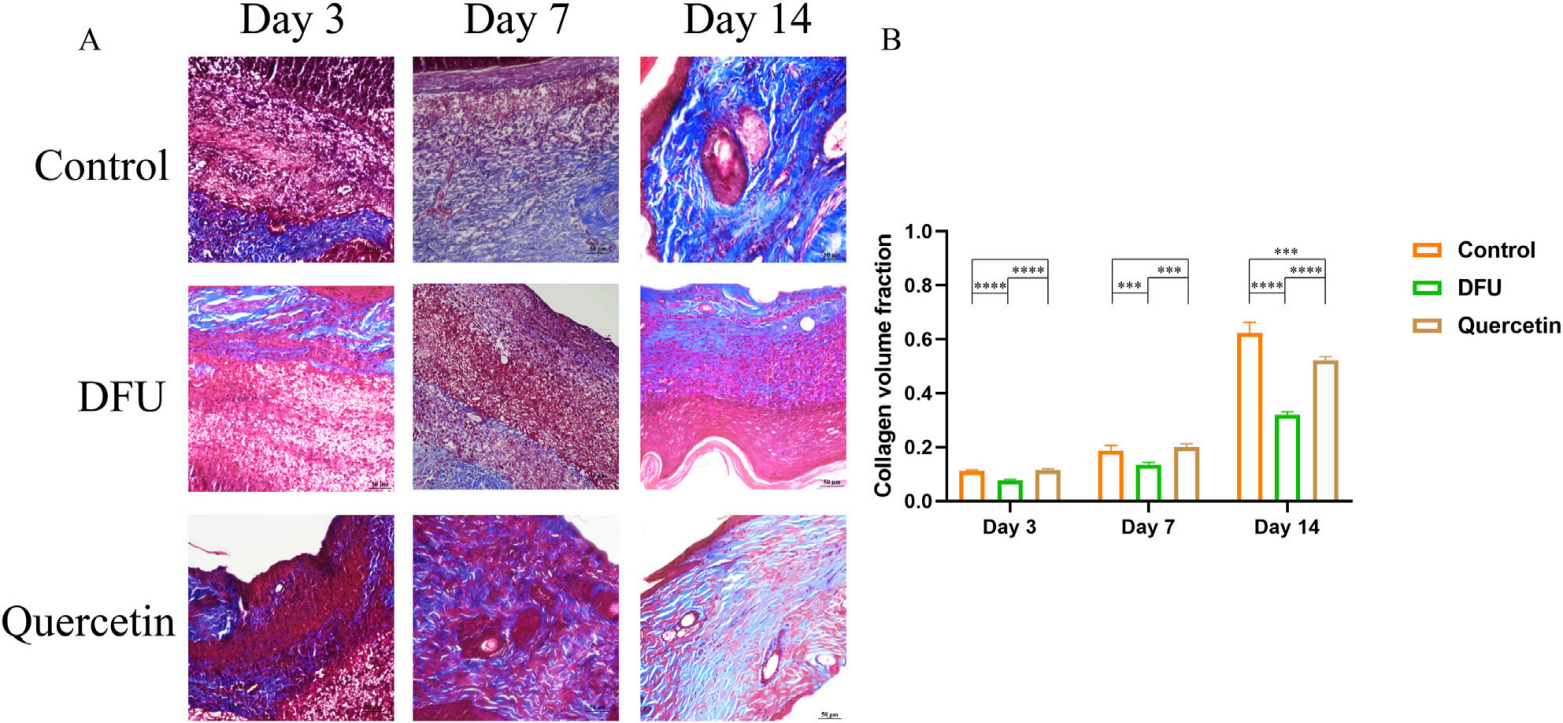
Masson staining (*P < 0.05, **P < 0.01, ***P < 0.005, and ****P < 0.001). **(A)** Masson staining; **(B)** Volume analysis of collagen.

The results of hematoxylin and eosin (HE) staining are presented in Figure 10. In the model group, observations on the third day of intervention revealed prominent red blood cells, hemorrhagic zones, marked edema, infiltration of inflammatory cells, a limited proliferation of disorganized fibroblasts, and an absence of new capillaries. By day 14, inflammatory cell infiltration persisted, along with a small number of fibroblasts, sparse collagen fibers, and a few new capillaries. In contrast, the treatment group exhibited minimal inflammatory cell infiltration, fibroblast proliferation, loose collagen fibers, and capillary neovascularization by the seventh day. By day 14, there was a significant reduction in inflammatory cells, the presence of mature fibroblasts, numerous dense collagen fiber bundles, a more mature epidermis, and the presence of most skin appendages. Masson staining results, depicted in Figure 11A, demonstrated that, compared to the collagen disorganization and loss observed in the model group, the collagen volume in the wound significantly increased following quercetin treatment (Figure 11B). These findings suggest that quercetin treatment substantially enhances wound healing in diabetic foot rats.

### 3.10 Immunofluorescence staining

**FIGURE 12:**
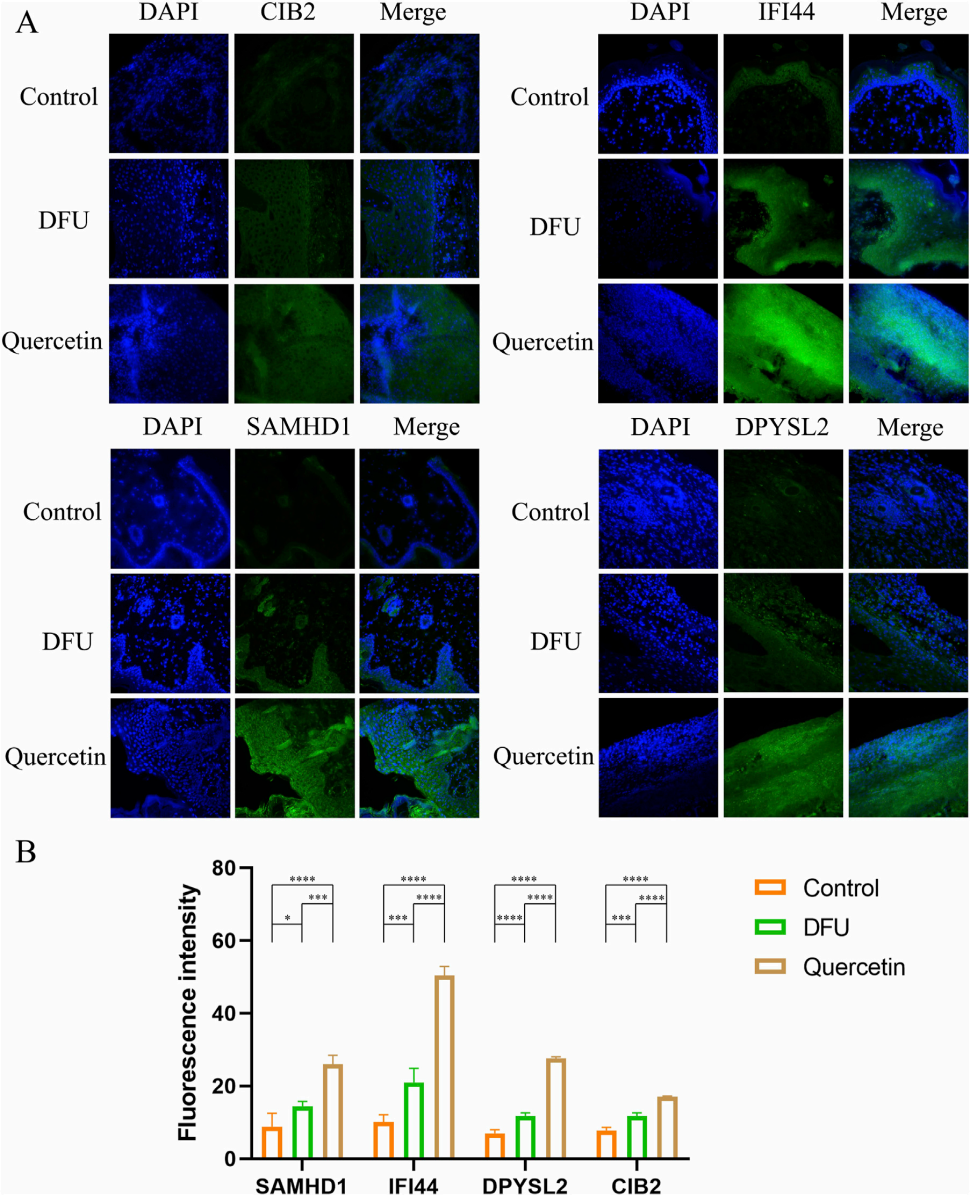
Immunofluorescence staining (*P < 0.05, **P < 0.01, ***P < 0.005, and ****P < 0.001). **(A)** Immunofluorescence diagram of each Hub gene on the seventh day; **(B)** Mean fluorescence intensity.

To corroborate the experimental findings, immunofluorescence staining was conducted on Hub genes within wound tissue collected on day 7 (Figure 12A). The analysis revealed that the fluorescence intensity of the target proteins was markedly elevated following quercetin treatment compared to the model group (Figure 12B), indicating a significant upregulation in the expression of these proteins post-treatment.

## 4 Discussion

Wound healing is a complex process that can be categorized into four distinct stages: hemostasis, inflammation, proliferation, and remodeling. These stages are intricately connected, and prolonged inflammation can negatively impact subsequent tissue regeneration (Martin and Nunan, 2015). In the context of diabetic wounds, hyperglycemia and the vascular damage it induces can extend the inflammatory phase, thereby impeding the healing process (Morton and Phillips, 2016). Such delays in wound healing may lead to Ricco et al. (2013) complications such as wound infection and destruction of the vascular bed, further exacerbating the healing challenges and creating a deleterious feedback loop. For patients with diabetes, it is imperative to interrupt this cycle promptly. Research indicates that quercetin possesses significant anti-diabetic (Dhanya, 2022), anti-inflammatory (Yuan et al., 2020), and circulatory-enhancing properties (Larson et al., 2010). Additionally, flavonoids have been demonstrated to modulate macrophage activity, thereby facilitating the repair of diabetic wounds (Fu et al., 2020). In this study, we explored the potential of puerarin to enhance wound healing in diabetic mice through the modulation of macrophages. The study yielded the following conclusions: (1) The topical application of puerarin effectively promotes wound healing in diabetic foot rats; (2) Quercetin facilitates wound healing by modulating the expression of CIB2, IFI44, DPYSL2, and SAMHD1; (3) Bioinformatics analysis suggests that quercetin’s regulation of DPYSL2 and SAMHD1, which contributes to wound healing, may occur through the modulation of macrophage activity. These findings provide a significant theoretical foundation for the treatment of refractory skin wounds in patients with diabetic foot.

Macrophages constitute a heterogeneous cell population that can be categorized into the pro-inflammatory M1 phenotype and the pro-reparative M2 phenotype. Prior research has demonstrated that M1 macrophages are pivotal in the initial stages of wound healing, primarily through the production of elevated levels of pro-inflammatory cytokines and the facilitation of a sustained inflammatory response (Qing et al., 2019). Conversely, the polarization of M2 macrophages has been associated with the enhancement of tissue repair processes. Increasing evidence suggests that M2 macrophages play a regulatory role in collagen production, myofibroblast differentiation, fibroblast regeneration, and angiogenesis during wound healing (Wynn and Vannella, 2016; Snyder et al., 2016). Furthermore, several studies have indicated that the depletion of M2 macrophages leads to a downregulation of growth factor levels during the proliferative phase of wound healing (Sica et al., 2015; Okizaki et al., 2015). Furthermore, increased concentrations of proinflammatory mediators (Mirza et al., 2015), including inducible nitric oxide synthase (iNOS) and interleukin-1 beta (IL-1β), are correlated with non-healing wound phenotypes. Chronic wounds are frequently characterized as being “stalled” in the inflammatory phase, which is linked to an impeded transition from M1 to M2 macrophage phenotypes during the later stages of wound healing.

This study employed machine learning techniques to identify four key genes—DPYSL2, CIB2, IFI44, and SAMHD1—that potentially play a significant role in the pathogenesis and progression of diabetic foot ulcer (DFU) disease. Through immune infiltration and single-cell omics analyses, it was determined that DPYSL2 and SAMHD1 may influence DFU development by modulating macrophage activity. Furthermore, quercetin may facilitate the healing of DFU wounds by modulating DPYSL2 and SAMHD1, thereby affecting macrophage function.

DPYSL2, or dihydropyrimidinase-like 2, encodes a member of the collapsing protein response mediator protein family. Folding reaction mediator proteins are capable of forming both homologous and heterotetrameric complexes, thereby facilitating neuronal guidance, growth, and polarity. The protein encoded by DPYSL2 is instrumental in promoting microtubule assembly and is essential for Sema3A-mediated growth cone collapse. Additionally, it plays a significant role in synaptic signaling through its interactions with calcium channels. The regulation of DPYSL2 is closely associated with the mTOR signaling pathway, which is known to promote cell growth and division (Miloslavski et al., 2014; Patursky-Polischuk et al., 2009), regulate macrophage polarization (Guertin and Sabatini, 2007; Efeyan and Sabatini, 2010), and is widely implicated in cancer biology. Analyses of immune infiltration and single-cell omics have indicated a correlation between DPYSL2 and macrophage activity. Furthermore, findings from foundational experiments provide preliminary support for these bioinformatics results, suggesting that the dysregulation of macrophages in diabetic foot ulcers (DFU) may be linked to alterations in DPYSL2 expression.

Macrophages, as central effector cells of innate immunity, critically rely on spatiotemporally regulated calcium ion (Ca^2+^) channel-mediated signaling dynamics to coordinate the initiation, execution, and resolution of immune responses (Feske et al., 2015). Upon pathogen recognition via pattern recognition receptors, macrophage activation triggers phospholipase C (PLC)-dependent inositol trisphosphate (IP3) generation, which mobilizes endoplasmic reticulum Ca^2+^ stores through IP3 receptors. Subsequent store depletion activates store-operated calcium entry (SOCE) via STIM1/Orai1 complexes (Dahiya et al., 2021), driving sustained extracellular Ca^2+^ influx that potentiates NF-κB and MAPK pathway activation, thereby inducing transcription of pro-inflammatory cytokines. During phagocytosis, localized Ca^2+^ transients mediated by channels such as TRPM2 regulate pseudopod extension and phagosome formation, while calmodulin (CaM)-dependent Ca^2+^ signaling facilitates lysosomal fusion for pathogen degradation. Calcium signaling further orchestrates macrophage polarization: the pro-inflammatory M1 phenotype relies on SOCE-NFAT axis activation to amplify inflammatory mediators (Liu et al., 2024; Liu et al., 2025), whereas the anti-inflammatory M2 phenotype may engage TRPV4-STAT6 pathways to promote tissue repair (Sumioka et al., 2023). Dysregulation of this balance underlies pathologies such as atherosclerosis and immunodeficiency disorders. Therapeutic strategies targeting these pathways are emerging, including SOCE inhibitors for autoimmune diseases, TRP channel modulators to skew polarization toward M2 phenotypes in fibrosis, and STIM1/Orai1 antagonists in clinical trials (Wulff et al., 2019). Collectively, Ca^2+^ channels serve as molecular hubs governing macrophage functions—from pathogen clearance to inflammatory homeostasis—making them pivotal targets for precision therapies in infection, cancer, and immune-metabolic disorders (Lee et al., 2024; Chen et al., 2021; Vera et al., 2023; Morotti et al., 2022; Nguyen et al., 2017; Grimaldi et al., 2016).

SAMHD1, or SAM domain and HD domain-containing protein 1, is an evolutionarily conserved innate immune molecule prevalent in both eukaryotic and prokaryotic organisms. It serves as a potent restriction factor for deoxynucleotide triphosphate hydrolase and human immunodeficiency virus type 1 (HIV-1), and it plays a crucial role in DNA damage repair and innate immune responses. Additionally, SAMHD1 is implicated in the regulation of macrophagepolarization (Li et al., 2022). Bioinformatics analyses have demonstrated that the expression levels of SAMHD1 mRNA are significantly reduced in patients with diabetic foot ulcers (DFU) compared to healthy individuals, a finding corroborated by experimental data. This suggests that SAMHD1 may be a therapeutic target of quercetin in the treatment of DFU. Immune infiltration analysis revealed a positive correlation between SAMHD1 expression and both M1 and M2 macrophages. Furthermore, single-cell analysis indicated that SAMHD1 is involved in macrophage regulation, implying that quercetin might facilitate DFU wound healing by modulating macrophage polarization via SAMHD1.

This study underscores the multifaceted role of macrophages in DFU pathogenesis and positions quercetin as a promising candidate for modulating macrophage polarization. Beyond diabetic wounds, these findings may inform therapeutic strategies for other chronic inflammatory conditions characterized by dysregulated macrophage activity, such as atherosclerosis, rheumatoid arthritis, or non-healing surgical wounds. For example, the IL-17 and PI3K/Akt pathways, which were enriched in our analysis, are also implicated in autoimmune disorders, suggesting that quercetin’s pleiotropic effects could be harnessed for broader applications. The integration of multi-omics and machine learning approaches demonstrates a robust framework for identifying novel therapeutic targets in complex diseases. This methodology could be adapted to explore other understudied pathologies, such as pressure ulcers or burn injuries, where macrophage dysfunction plays a critical role. Furthermore, combining transcriptomic data with proteomic or metabolomic profiling may uncover additional layers of regulation, such as post-translational modifications or metabolic rewiring, that influence quercetin’s mechanism of action (Bowen et al., 2024). Future research should prioritize elucidating the precise molecular pathways through which quercetin regulates SAMHD1 and DPYSL2. CRISPR-based gene editing or siRNA knockdown experiments could validate the causal roles of these genes in macrophage polarization and wound healing. Additionally, investigating quercetin’s synergistic effects with existing therapies—such as antibiotics (to combat *Staphylococcus aureus* infection) or recombinant growth factors—may optimize clinical outcomes. For instance, combination therapies could mitigate biofilm formation while enhancing tissue regeneration. Long-term toxicity studies and explorations of alternative delivery methods are also critical to address bioavailability challenges and ensure patient safety. Quercetin’s poor water solubility and rapid metabolism often limit its therapeutic efficacy, necessitating innovative formulations to improve its pharmacokinetic profile.

While our findings highlight quercetin’s therapeutic potential, certain limitations inherent to animal models must be acknowledged. The STZ-induced diabetic rat model primarily mimics type 1 diabetes, whereas human diabetic foot ulcers (DFUs) predominantly arise in type 2 diabetes patients. Differences in metabolic regulation, immune responses, and wound healing kinetics between rodents and humans may influence the translatability of these results. Additionally, the study utilized male rats exclusively, omitting potential sex-specific variations in immune function or drug response. To bridge this gap, future studies should incorporate type 2 diabetes models, such as high-fat diet-fed rodents, and include both sexes to enhance clinical relevance. While our study provides mechanistic insights into quercetin’s role in DFU healing, future work should prioritize human-relevant models. For instance, ulcerated skin explants from diabetic patients cultured *ex vivo* could be treated with quercetin to assess SAMHD1/DPYSL2 expression and macrophage polarization. Such models (Kubilus et al., 2004) better recapitulate the diabetic microenvironment and would validate our findings in a translational context. Notably, quercetin is currently regulated as a dietary supplement in many regions, necessitating rigorous pharmacokinetic studies and standardized formulations before clinical trials. Future research should explore its bioavailability, optimal dosing, and safety profiles in diabetic populations to facilitate its transition from a nutraceutical to a therapeutic agent (Williamson et al., 2018; Andres et al., 2018).

## 5 Conclusion

In summary, this study advances our understanding of quercetin’s role in DFU healing and highlights macrophage modulation as a key mechanism. By addressing the aforementioned limitations and expanding research horizons, these insights could pave the way for innovative, targeted therapies to alleviate the global burden of diabetic complications.

## Data Availability

The datasets presented in this study can be found in online repositories. The names of the repository/repositories and accession number(s) can be found in the article/supplementary material.
